# The relations between personality, components of executive functions, and intelligence in children and young adults

**DOI:** 10.1007/s00426-021-01623-1

**Published:** 2021-12-21

**Authors:** Verena E. Johann, Julia Karbach

**Affiliations:** 1 Department of Psychology, University of Koblenz-Landau, Fortstraße 7, 76829 Landau in der Pfalz, Germany; 2 Center for Research on Individual Development and Adaptive Education of Children at Risk (IDeA), Frankfurt, Germany

## Abstract

Previous studies in adults showed heterogeneous results regarding the associations of personality with intelligence and executive functions (EF). In children, there is a lack of studies investigating the relations between personality and EF. Therefore, the aim of our study was to examine the relations between the Big Five personality traits, EF, and intelligence in a sample of children (Experiment 1) and young adults (Experiment 2). A total of 155 children (Experiment 1, mean age = 9.54 years) and 91 young adults (Experiment 2, mean age = 23.49 years) participated in the two studies. In both studies, participants performed tasks measuring working memory (WM), inhibitory control, cognitive flexibility, and fluid intelligence and completed a personality questionnaire. In Experiment 1, we found a negative relation between neuroticism and intelligence. In Experiment 2, we found a positive relation between conscientiousness and intelligence and a positive relation between conscientiousness and cognitive flexibility. Our results suggest a complex interplay between personality factors, EF, and intelligence both in children as well as in young adults.

## Introduction

Executive functions (EF) describe higher-level cognitive control processes supporting the adaption to continuous changes in the environment. According to Miyake et al. (2000), there are three moderately correlated but clearly separable EF: monitoring and updating of working memory representations (updating or working memory, WM), inhibition of prepotent or irrelevant information and action tendencies (inhibition) and switching between different tasks or representations (cognitive flexibility or shifting). EF have been shown to be essential for mental and physical health, success in school and in life, and cognitive, social, and psychological development (Diamond, 2013).

In children, the structure of EF appears to be unitary during the preschool years (for a review, see Karr et al., 2018). Studies with primary school children also reported a structure with a common EF factor (Brydges et al., 2012; Xu et al., 2013) or two EF factors (Lee et al., 2012). Up to the age of about 10 years, the structure of EF seems to be differentiated and most studies reported models with three EF factors (Duan et al., 2010; Lehto et al., 2003; Rose et al., 2012; Shing et al., 2010; Wu et al., 2011).

Given the importance of EF, much recent work focused on investigating the relations among various EF and their relations with intelligence (Friedman et al., 2006). In adults, WM and updating are considered strong predictors of intelligence, whereas shifting and inhibition seem to be unrelated to intelligence (Benedek et al., 2014; Friedman et al., 2006). Moreover, there is evidence that a common EF factor is associated with intelligence in young children (7- to 9-year-olds, Brydges et al., 2012). In older children, WM, inhibition, and shifting were related to intelligence (Duan et al., 2010; van der Sluis et al., 2007). The relation between a common EF factor and intelligence in young children might stem from a genetic overlap between EF and intelligence. Engelhardt et al. (2016) found that genetic influences on a common EF factor accounted for a large proportion of phenotypic variance and all of the genetic variance in intelligence in 7- to 15-year-old twins.

In contrast, little is known about the relations between EF and personality, although different observations suggest an association. First, EF and personality have been linked to similar brain structures, mainly in the prefrontal cortex (DeYoung et al., 2010; Fuster, 2000; Wagner et al., 2005). For instance, the dorsolateral prefrontal cortex is associated with extraversion (DeYoung et al., 2010), neuroticisms, conscientiousness (Kapogiannis et al., 2013) and agreeableness (Koelsch et al., 2013). Moreover, there are studies showing that the dorsolateral prefrontal cortex was engaged in response inhibition (Nee et al., 2007; Wagner et al., 2005), switching (Kim et al., 2012; Wagner et al., 2005), and in all forms of WM toward a goal (Fuster, 2000; Owen et al., 2005).

Second, there is a bulk of evidence suggesting that personality disorders are attended by deficits in EF. For instance, patients with antisocial personality disorder suffered from deficits in planning ability, set shifting, response inhibition, and visual memory (Dolan & Park, 2002). In patients with borderline personality disorder, impaired WM (Ruocco, 2005; Stevens et al., 2004) as well as deficits in attention, cognitive flexibility, planning, speeded processing, and visuospatial abilities were found (Ruocco, 2005).

The observations that similar brain structures are associated with personality traits and EF and that personality disorders are often related to deficits in EF indicate a relation between personality and EF. However, evidence for associations between the Big Five personality traits and WM, inhibition, and cognitive flexibility in adults is heterogeneous (see below). Regarding children, there is only one study in which the associations between an EF factor and the Big Five personality traits were investigated (Neuenschwander et al., 2013).

There is also evidence for relations between personality and intelligence in adults (see below). Although there is a large number of studies investigating these relations, a theoretical integration of cognitive abilities and personality factors remained largely unaddressed. Chamorro-Premuzic & Furnham (2004) provided a conceptual framework for understanding the links between personality and intelligence. It conceptualizes three different levels of intelligence (intellectual ability, intelligence test performance and subjectively assessed intelligence), as well as the Big Five personality traits. Intellectual ability refers to ability as trait (‘actual’ intelligence), while cognitive or intelligence test performance refers to it as output. Despite this distinction, the authors stated that established intelligence tests are reasonably good indicators of a person’s intellectual ability. Moreover, they assume that most variance in intelligence performance is accounted for by intelligence. Nevertheless, it has been argued that personality factors affect test performance rather than actual intelligence. A negative association between neuroticism and intelligence scores could thereby be explained by higher anxiety and stress during test situations. A positive relation between extraversion and intelligence could be explained by higher arousal or speed of response. In case that personality factors rather affect cognitive test performance than cognitive abilities, performance on EF tasks could be influenced in a similar way. However, it is also possible that personality factors might influence the development of intellectual skills (and vice versa). Chamorro-Premuzic & Furnham (2004) and Ackerman (1999) propose that some personality factors might influence the development of cognitive abilities. Especially openness and conscientiousness could determine whether an individual engages in intellectually beneficial activities. Thus, personality traits might directly influence intelligence and even EF. By contrast, it is also possible that actual cognitive abilities influence the development of personality factors. Although there are some studies on the relations between personality factors and EF in adults and a large number of studies investigating the relations between personality factors and intelligence, the results are still heterogeneous. Moreover, there is a lack of studies examining the relations between personality factors, EF, and intelligence in children.

### The relations between personality and EF

Regarding WM, there are studies demonstrating better WM performance in individuals with higher levels of extraversion (Campbell et al., 2011; Gray & Braver, 2002; Lieberman, 2000). Dima et al. (2015) found that neuroticism and conscientiousness respectively constrained and facilitated brain connectivity within a WM network comprising the dorsolateral prefrontal, parietal, and anterior cingulate cortex. In contrast, extraversion was not relevant to task-dependent effective connectivity. In another study (DeYoung et al., 2009) the relation between the Big Five personality trait Openness/Intellect, which is composed of openness to experience and intellect, and WM was investigated. The authors reported that intellect but not openness was positively correlated with WM accuracy and with accuracy-related brain activity. In contrast, there are also studies reporting no relations between personality traits and WM performance (Fleming et al., 2016; Unsworth et al., 2009).

Regarding inhibition, a study by Haas et al. (2006) demonstrated that extraversion was positively correlated with the interference effect in an emotional Stroop task. In another study (Fleming et al., 2016), extraversion was not related to performance on tasks measuring response inhibition or interference control (antisaccade task, stop-signal task, and Stroop task; see also Avila & Parcet, 2001). However, neuroticism was negatively correlated with performance on the antisaccade task and openness was negatively correlated with performance on the stop-signal task. There are also studies showing that neuroticism was not associated with inhibition abilities (Avila & Parcet, 2001; Ettinger et al., 2005; Unsworth et al., 2009).

Regarding the relation between personality and cognitive flexibility, Campbell et al. (2011) found that introverted participants performed better on a set shifting task than extraverted individuals. This finding is in line with the result from Umemoto and Holroyd (2016) who found that performance on a switching task was worse for participants high in extraversion, agreeableness and neuroticism. In contrast, Smillie et al. (2009) found that psychoticism but neither extraversion nor neuroticism predicted performance on a Winsconsin Card Sorting Test. Fleming et al. (2016) showed by means of structural equation modeling that conscientiousness was positively associated with mental set shifting. In another study (Unsworth et al., 2009), a significant latent correlation between openness and a fluency factor was found.

Whereas these studies were conducted with young or older adults, evidence for a relation between personality traits and EF in children is rare. Neuenschwander et al. (2013) reported positive correlations between an EF factor and emotional stability, conscientiousness, and culture/openness in children attending grade 1 and 2.

### The relations between personality and intelligence

In adults, the most consistent finding regarding the relation between intelligence and personality is that intelligence is negatively correlated with neuroticism (Ackerman & Heggestad, 1997; Furnham et al., 1998; Moutafi et al., 2003) and positively correlated with openness to experience (Austin et al., 2002; Chamorro-Premuzic et al., 2005; Moutafi et al., 2003; Zeidner & Matthews, 2000). Regarding extraversion, the relation with intelligence has sometimes been positive (e.g.Ackerman & Heggestad, 1997; Soubelet & Salthouse, 2011) and sometimes negative (e.g.Austin et al., 2002; Moutafi et al., 2005). There are also contradictory results on the relation between conscientiousness and intelligence. Some studies reported negative relations (Moutafi et al., 2003, 2004, 2006a, 2006b), whereas other studies found no relation or even positive relations (Ackerman & Heggestad, 1997; Booth et al., 2006; Kyllonen, 1997). Agreeableness was consistently unrelated to intelligence (Ackerman & Heggestad, 1997; Austin et al., 2002; Kyllonen, 1997).

There are only a few studies investigating the relation between personality and intelligence in children and adolescents. Demetriou et al. (2003) found that cognitive performance was weakly related to openness and conscientiousness in 12- to 17-year-olds. Di Blas and Carraro (2011) investigated children aged between 8 and 11 years and showed by means of regression analyses that higher fluid intelligence was significantly associated with parent-reported lower extraversion and higher imagination. Moreover, the conscientiousness facets orderliness and perseverance showed antagonist relations with intelligence whereas the emotional stability facet self-confidence was positively related with children’s intelligence scores.

In contrast, Asendorpf and Van Aken (2003) reported modest correlations between children’s intelligence and their teachers’, parents’, and friends’ ratings on culture levels, across all ages, whereas conscientiousness uniquely predicted IQ at ages 4–6 and 10 and neuroticism predicted IQ at age 10 and 12.

### Summary

In sum, there are different observations suggesting relations between personality traits and EF. However, studies investigating these associations led to inconsistent results in adults. In children, there is only one study reporting positive correlations between an EF factor and emotional stability, conscientiousness, and culture/openness (Neuenschwander et al., 2013). Regarding the relation between personality and intelligence, several studies reported associations of neuroticism, conscientiousness, and openness with intelligence, but there are also contradictory results. In children, there are only a few studies with heterogeneous findings. These inconsistencies could be explained by different research methods such as different tasks assessing EF and intelligence or different personality questionnaires. Moreover, some studies investigated the relations between personality factors and cognitive functioning by means of functional magnetic resonance imaging (fMRI) with differences in activation or functional connectivity as indicator for cognitive performance (e.g.Dima et al., 2015; Gray & Braver, 2002). In contrast, behavioral studies investigated the relations between personality and performance on EF or intelligence tasks (e.g.Campbell et al., 2011; Fleming et al., 2016).

### Research questions

The main aim of this study was to investigate associations of personality traits with WM, inhibition, cognitive flexibility, and intelligence in children (Experiment 1) and young adults (Experiment 2). We focused on the Big Five personality traits, which have a slightly different connotation in children. The questionnaire applied in Experiment 1 comprised the five scales extraversion, neuroticism, conscientiousness, benevolence (instead of agreeableness) and imagination (instead of openness to experience). In children (Experiment 1), we expected positive relations between imagination, conscientiousness, and intelligence (Demetriou et al., 2003; Di Blas & Carraro, 2011). Moreover, we expected a negative relation between neuroticism and intelligence (Asendorpf & Van Aken, 2003). We had no specific hypotheses regarding the relations between personality and EF.

In adults (Experiment 2), we expected neuroticism to be negatively related to intelligence (Moutafi et al., 2003) and openness to be positively related to intelligence (Moutafi et al., 2003). Due to heterogeneous previous results, we had no specific hypotheses regarding the relations between personality and EF.

## Material and methods

### Participants

#### Experiment 1

155 elementary school students (mean age = 9.54 years, SD = 0.50; age range  9–10 years; 38.7% female) participated in the study. Children attended Grade 3 or 4 and were recruited via flyers in primary schools. They received 10€ as compensation for their participation in the study. Parents and children provided written informed consent. Moreover, parents provided information on their highest educational achievement. Most of them (79%) stated to have reached a high school diploma (“Abitur”) or a higher educational level.

#### Experiment 2

91 young adults (mean age = 23.49 years, SD = 3.21; age range 18–31 years; 57.1% female) participated in the study. Participants provided written informed consent and demographic information. Most of them were students at a university (95.6%). Participants were recruited via advertisements posted on campus and distributed through a university mailing list and they received 10€ as compensation for their participation in the study.

### Materials and procedure

The procedure was the same for both experiments. The studies comprised one session, in which all participants performed a complex span task (WM), a child-friendly Stroop-like task (Experiment 1) or a flanker task (Experiment 2) (inhibition), task switching (flexibility), a fluid intelligence test, and a personality questionnaire. Participants in both experiments were tested at the laboratory. All tests were administered in small groups of up to three participants.

### Tasks

#### Experiment 1

All EF tasks were implemented by Synaptikon GmbH and provided online via www.neuronation.com. Details of all tasks and questionnaires are provided in Appendix A.

##### WM: Complex span task (cf. Karbach et al., 2015)

In the complex span task, participants recalled the sequence of pictures (dinosaurs) against a background processing decision task (deciding if a star or a moon was presented). The dependent variable was the accuracy (% correct, ACC).

##### Inhibition: Stroop-like task (cf. Borella et al., 2010)

In the Stroop-like task, children saw a series of fruits (pear, lemon, strawberry, orange) in which the congruency between the upper part and the lower part was manipulated. In congruent trials, the upper part matched the lower part, in incongruent trials, there was no match (e.g., upper part of a lemon on a lower part of a pear). Participants were to inhibit the upper part of the picture and indicate what kind of fruit they saw based on the lower part of the picture as quickly as possible by pressing one of four response buttons (A, S, K, L) on a computer keyboard. As dependent variable served the interference effect, calculated as the difference in performance between congruent and incongruent trials based on ACC.

##### Flexibility: task switching (cf. Karbach & Kray, 2009)

Participants were instructed to perform two tasks A and B in mixed-task blocks (switching tasks on every second trial). Task A required participants to decide whether a picture showed a fruit or a vegetable and task B whether a picture was small or large. The same two response keys were used for both task sets. This design allows calculating switching costs, which means the difference in performance between switch trials and stay trials (trials not requiring to switch the task) based on ACC representing the ability to flexibly switch between tasks.

##### Fluid intelligence: Raven colored progressive matrices (Raven et al., 2001)

In the Raven’s task, participants selected one of six figures that best completed a pattern. The task comprised set A and set AB, each with 12 items. Three items were applied as practice items, followed by up to 21 test items increasing in difficulty. The task was aborted after 10 min. The test score was the ACC.

##### Personality: HiPIC-30 (Bleidorn & Ostendorf, 2009; Vollrath et al., 2016)

Self-reported personality was assessed by the German version of the hierarchical personality inventory for children (HiPIC-30, Vollrath et al., 2016). Translations of the items were extracted from the German long version (Bleidorn & Ostendorf, 2009). Participants rated 30 items on a Likert scale from 1 („I don’t agree at all “) to 5 („I agree completely “). The questionnaire included six items referring to neuroticism, imagination, conscientiousness, benevolence, and extraversion, respectively. The dependent variables were the mean scores on the five personality scales.

#### Experiment 2

All EF tasks and the intelligence test were programmed with the experimental software E-Prime 2.0. Details of all tasks and questionnaires are provided in Appendix B.

##### WM: counting span task (cf. Kane et al., 2004)

In the counting span task, participants recalled digits against a background counting task. Participants were to count the number of dark blue circles in each display and repeat the total number. At the recall cue, participants had to recall the respective numbers of dark blue circles in the correct order. The dependent variable was the ACC of recalled sets.

##### Inhibition: Flanker task (cf. Eriksen & Eriksen, 1974)

In the flanker task, participants were presented with a sequence of stimuli consisting of five letters and were instructed to press the correct key according the central stimulus (target) while ignoring the four stimuli on the left and the right side (distractors). The central target letter was either an “H” or an “S” and was surrounded by two “H” or two “S” on the left and the right side. The response interference effect was calculated as the difference in performance between congruent (SSSSS, HHHHH) and incongruent (SSHSS, HHSHH) trials based on ACC.

##### Flexibility: task switching (cf. Karbach & Kray, 2009)

The switching task was the same as described in Experiment 1.

##### Fluid intelligence: Raven advanced progressive matrices (Raven et al., 1998)

In the Raven’s task, participants selected one of eight figures that best completed a pattern. They first performed three practice items, followed by up to 36 test items increasing in difficulty. The task was aborted after 20 min. The test score was the ACC.

##### Personality: BFI-S (Gerlitz & Schupp, 2005)

Self-reported personality was assessed by the BFI-S (Gerlitz & Schupp, 2005) which is a German 15-item version based on the original 44-item Big Five Inventory (BFI; John et al., 1991). The BFI-S assesses the Big Five personality traits by means of three items per dimension. Participants rated these statements on a 7-point scale ranging from 1 (“does not apply to me at all”) to 7 (“applies to me perfectly”). Item selection and construction is described in detail by Gerlitz and Schupp (2005). The dependent variables were the mean scores on the five personality scales.

### Data analyses

The analyses for Experiment 1 and 2 were based on correlations and two structural equation models (SEM), which were calculated with Mplus 7.4, using standard maximum likelihood estimation (ML). In line with Beauducel and Wittmann (2005), model-fit was evaluated with the *χ*^2^-test, the comparative fit index (CFI), the root mean square error of approximation (RMSEA), and the standardized root mean square residual (SRMR). For model identification, the first loading of a latent factor was fixed to 1. We considered all *p* values below 0.05 to be significant. In order to increase the reliability of the personality scales, three items of the BFI-S (one item of the scales conscientiousness, extraversion, and neuroticism, respectively) and four items of the HiPIC-30 (one item of the scales conscientiousness, extraversion, benevolence, and neuroticism, respectively) were excluded from the analyses.

## Results

**Fig. 1:**
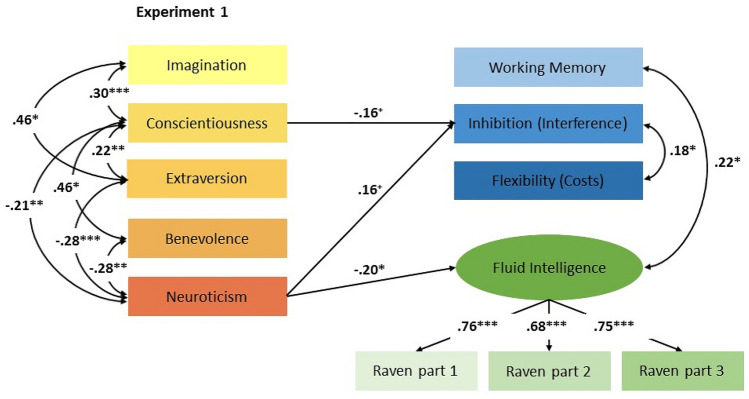
The relations of personality to EF and intelligence in children. The fit of the model to the data was excellent [*X*^2^(*df* = 16) = 13.39, *p* = 0.64; CFI = 0.99; RMSEA = 0.01 (90% CI 0.01–0.06); and SRMR = 0.02]. Non-significant paths from the personality factors to EF and fluid intelligence are not displayed. All parameters are standardized. The squares represent observed variables and the circles represent latent variables. ^+^*p* < 0.06, *p < 0.05, *p < 0.01, ***p < 0.001

**Fig. 2:**
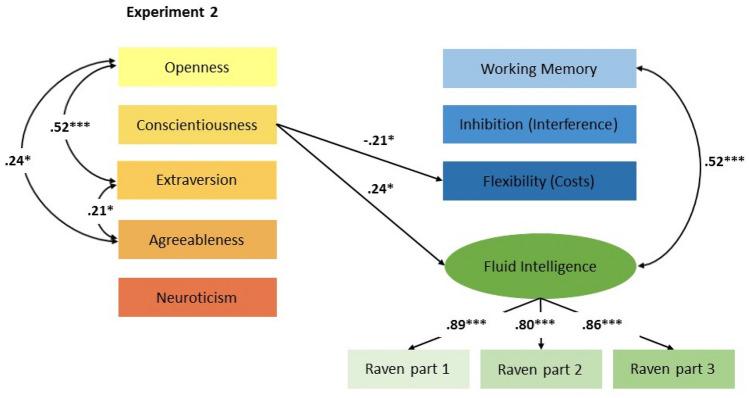
The relations of personality to EF and intelligence in young adults. The fit of the model to the data was good [*X*^2^(*df* = 16) = 22.00, *p* = 0.14; CFI = 0.97; RMSEA = 0.06 (90% CI 0.01–0.12); and SRMR = 0.03]. Non-significant paths from the personality factors to EF and fluid intelligence are not displayed. All parameters are standardized. The squares represent observed variables and the circles represent latent variables. *p < 0.05, **p < 0.01, ***p < 0.001

We report reliabilities for the personality measures, followed by correlations between personality, EF, and intelligence and two SEM on the relations between personality, EF, and intelligence. Analyses of the EF tasks were based on ACC (% correct). Practice blocks and the first trial in each block were not analyzed. Outliers were defined as cases with values more than 3 times the interquartile range (max = 3.2%) and were excluded from all analyses. Descriptive statistics for the personality scales, EF tasks, and the intelligence measure of Experiment 1 and 2 can be found in Table 1. The main findings are displayed in Fig. 1 (Experiment 1) and Fig. 2 (Experiment 2).

**Table 1:** Means (M) and standard deviations (SD) of the personality scales, the EF tasks, and the intelligence test

Scale/task	Dependent variable	*M* (SD)	*M* (SD)
		Experiment 1	Experiment 2
Imagination/openness		3.93 (0.60)	5.03 (1.05)
Conscientiousness		3.98 (0.59)	5.43 (0.90)
Extraversion		3.48 (0.56)	5.21 (1.27)
Benevolence/agreeableness		3.44 (0.64)	5.61 (0.75)
Neuroticism		2.44 (0.79)	4.34 (1.48)
Complex span task	ACC (%)	70.26 (13.64)	70.60 (23.89)
Stroop-like task/Flanker task	Interference effect (ACC, %)	12.32 (9.02)	7.42 (5.95)
Task switching	Switching costs (ACC, %)	9.66 (7.98)	2.06 (5.33)
Intelligence	ACC (%)	78.08 (14.34)	58.58 (17.26)

*ACC* accuracy

### Reliability

#### Experiment 1

We computed Cronbach’s alpha as measure for internal consistency. Reliability coefficients of the personality scales ranged from acceptable (Extraversion, *α* = 0.48) to good (Neuroticism, *α* = 0.74) (see Table 2).

**Table 2:** Reliabilities (Cronbach’s *α*) of the personality scales in Experiment 1 and 2

	Internal consistency	
	Experiment 1	Experiment 2
Imagination/openness	0.63	0.62
Conscientiousness	0.66	0.70
Extraversion	0.48	0.78
Benevolence/agreeableness	0.48	0.48
Neuroticism	0.74	0.70

#### Experiment 2

Reliability coefficients of the personality scales ranged from acceptable (agreeableness, *α* = 0.48) to good (extraversion, *α* = 0.78) (see Table 2).

### The associations of personality with EF and intelligence

#### Experiment 1

Correlations between the personality scales, EF tasks, and the intelligence measure of Experiment 1 can be found in Table 3. Inhibition interference was negatively correlated with imagination and conscientiousness, but positively correlated with neuroticism. These results indicate that higher values on imagination and conscientiousness were associated with higher inhibitory control, whereas higher values on neuroticism were associated with worse inhibitory control. WM performance was positively correlated with intelligence.

**Table 3:** Correlations of the personality scales, the EF tasks, and the intelligence test (Experiment 1/Experiment 2)

	1	2	3	4	5	6	7	8
1. Imagination/openness								
2. Conscientiousness	0.30***/0.05							
3. Extraversion	0.46***/0.52***	0.22**/0.04						
4. Benevolence/agreeableness	– 0.01/0.24*	0.34***/0.16	0.01/0.21*					
5. Neuroticism	– 0.11/– 0.04	– 0.21*/0.– 03	– 0.28***/0.02	– 0.28***/0.07				
6. Complex span task (ACC)	– 0.01/0.16	– 0.01/0.04	– 0.06/0.19	0.07/0.01	0.10/0.11			
7. Stroop-like task/Flanker task (Interference effect)	– 0.18*/– 0.12	– 0.24**/– 0.12	– 0.12/– 0.13	– 0.12/– 0.19	0.21**/0.06	0.01/– 0.11		
8. Task switching (Switching costs)	– 0.12/– 0.05	0.02/0.19	– 0.01/– 0.08	– 0.02/– 0.07	0.11/0.01	– 0.06/0.07	0.16/– 0.11	
9. Raven (ACC)	– 0.04/0.06	– 0.10/0.12	– 0.10/– 0.01	– 0.11/– 0.14	– 0.13/– 0.16	0.17*/0.47***	0.01/– 0.08	– 0.07/0.05

*ACC* accuracy

*p < 0.05, **p < 0.01, *** *p* < 0.001

The further analysis was based on a SEM with the dependent variables WM ACC, inhibition interference, switching costs, and fluid intelligence. Fluid intelligence was represented as a latent factor in the model which was well-defined with substantial and significant factor loadings, indicating systematic common variance. The five predictors were the personality factors imagination, conscientiousness, extraversion, benevolence, and neuroticism. The fit of the model to the data was excellent [*X*^2^(*df* = 16) = 13.39, *p* = 0.64; CFI = 0.99; RMSEA = 0.01 (90% CI = 0.01–0.06) and SRMR = 0.02]. The five predictors showed different relations with EF and intelligence. Neuroticism was negatively related to intelligence, which means that higher values on neuroticism were associated with poorer performance on the intelligence test. Thus, intelligence was predicted by neuroticism (Pseudo-*R*^*2*^ = 6%). There were no significant relations between personality factors and EF. However, there was a marginally significant positive relation between neuroticism and inhibition interference indicating that higher levels of neuroticism were associated with worse inhibitory control. In contrast, conscientiousness was marginally negatively related to inhibition interference indicating that higher levels of conscientiousness were associated with better inhibition ability. Thus, inhibition was predicted by neuroticism and conscientiousness (Pseudo-*R*^*2*^ = 9%).

Notably, the predictors were tested simultaneously, which means that each significant relation explained variance over and above the other predictors.

#### Experiment 2

Correlations between the personality scales, EF tasks, and the intelligence measure of Experiment 2 can be found in Table 3. There were no significant correlations except for a significant correlation between WM performance and intelligence. The further analysis was based on a SEM with the dependent variables WM ACC, inhibition interference, switching costs, and fluid intelligence. Fluid intelligence was represented as a latent factor in the model which was well-defined with substantial and significant factor loadings, indicating systematic common variance. The five predictors were the personality factors openness, conscientiousness, extraversion, agreeableness, and neuroticism. The fit of the model to the data was good [*X*^2^(*df* = 16) = 22.00, *p* = 0.14; CFI = 0.97; RMSEA = 0.06 (90% CI 0.01–0.12); and SRMR = 0.03]. The five predictors showed different relations with EF and intelligence. Conscientiousness was positively related to intelligence, which means that higher levels of conscientiousness were associated with higher performance on the intelligence test. Moreover, conscientiousness was negatively related to switching costs indicating that higher levels of conscientiousness were associated with better switching performance. Thus, both intelligence (Pseudo-*R*^*2*^ = 8%) and switching costs were predicted by conscientiousness (Pseudo-*R*^*2*^ = 5%). Notably, the predictors were tested simultaneously, which means that each significant relation explained variance over and above the other predictors.

## Discussion

The main aim of this study was to investigate associations of personality factors with WM, inhibition, cognitive flexibility, and intelligence in children (Experiment 1) and young adults (Experiment 2). In Experiment 1, we found a significant negative relation between neuroticism and intelligence in children, which was in line with our expectation. This result fits the findings from studies reporting negative relations between neuroticism and intelligence in children (Asendorpf & Van Aken, 2003) and adults (Ackerman & Heggestad, 1997; Furnham et al., 1998; Moutafi et al., 2003). It is also in line with findings on the relation between neuroticism and academic achievement since there is a bulk of evidence suggesting a negative relation between neuroticism and student’s grade point average (e.g., Chamorro-Premuzic & Furnham, 2003a, 2003b).

Against our expectation, neither imagination nor conscientiousness was associated with intelligence. These results could be explained by the fact that we used a self-report questionnaire whereas in studies reporting positive relations between openness or culture/intellect and intelligence (Asendorpf & Van Aken, 2003; Di Blas & Carraro, 2011) parents or peers rated personality factors. There is evidence that elementary school children are less proficient providing reliable information about their own personality traits (Laidra et al., 2007). Regarding the non-existent relation between imagination and intelligence, the different results could be explained by the slightly different connotation of the personality scale imagination in children, which comprises the subscales openness, intellect, and creativity. In the past, there was a debate about the interpretation of the openness factor, with some researchers (e.g., McCrae & Costa, 1997) defining openness by such characteristics as imaginative, curious and esthetically sensitive, whereas others (e.g., Goldberg, 1990) define it by intellectual characteristics. Divergent results could also be explained by differences in assessing intelligence. Di Blas and Carraro (2011) also assessed children’s nonverbal intelligence with the CPM, but they selected only items with proportions of correct responses in the range of 0.20 ≤ *p* ≤ 0.80. In contrast, Laidra et al. (2007) applied Raven’s Standard Progressive Matrices.

Regarding the relations between personality and EF, we had no hypotheses. Neuenschwander et al. (2013) reported positive correlations between a general EF factor and emotional stability, conscientiousness, and culture/openness in children attending grade 1 and 2. We found a marginally significant positive relation between neuroticism and inhibition interference indicating that higher levels of neuroticism were associated with worse inhibition. A significant zero-order correlation between neuroticism and inhibition supported this assumption (see Table 3) and is in line with the results from Neuenschwander et al. (2013). Moreover, there was a marginally significant negative relation between conscientiousness and inhibition interference indicating that higher levels of conscientiousness were associated with better inhibition ability. A significant zero-order correlation also supported this result und fits the finding from Neuenschwander et al. (2013). In contrast, there was only a significant relation between imagination and the inhibition based on zero-order correlations. WM and cognitive flexibility were unrelated to personality factors in children. Heterogeneous results might be caused by the fact that in the study from Neuenschwander et al. (2013), parents rated personality factors of their children whereas we applied a self-report questionnaire.

The result that neuroticism was negatively related to intelligence could indicate that higher levels of neuroticism negatively influenced performance on the intelligence task (possibly because of higher performance anxiety). It is also possible that higher levels of neuroticism negatively affect the development of intelligence or vice versa, but these assumptions need to be tested in longitudinal designs.

In Experiment 2, there was no relation between openness and intelligence. This result was against our expectation and previous results (Moutafi et al., 2003). However, Moutafi et al. (2006a, 2006b) showed that only two of six facts (“Ideas” and “Actions”) of the NEO PI-R were related to fluid intelligence. The questionnaire we applied did not comprise the facet “Action” which could explain the non-significant relation between openness and intelligence. Moreover, there is evidence that openness correlated more strongly with verbal/crystallized intelligence than with EF and fluid intelligence (Schretlen et al., 2010).

We also found a significant relation between conscientiousness and intelligence. This result is line with previous studies (Ackerman & Heggestad, 1997; Kyllonen, 1997). However, there is also evidence that conscientiousness is negatively related to intelligence (Moutafi et al., 2003, 2004, 2006a, 2006b)). Heterogeneous results could also be explained by the sample composition. In our study, most of the participants were university students. Therefore, the variance in intelligence and conscientiousness might have been restricted. Moreover, Moutafi et al. (2006a, 2006b) showed that only three of six facets of conscientiousness (“Order”, “Self-Discipline”, and “Deliberation”) were related to intelligence. Therefore, inconsistent findings might also be explained by questionnaires with different scales. Di Blas and Carraro (2011) showed that only two facets of conscientiousness (“Orderliness” and “Perseverance”) accounted for significant unique proportions of IQ variability. The two facets showed antagonistic associations, with “Perseverance” being related positively, but “Orderliness” negatively with intelligence performance. Thereby the relation between conscientiousness and intelligence might be strongly influenced by the focus of the specific personality questionnaire.

Regarding the relation between personality and EF, we only found a significant negative relation between conscientiousness and switching costs, indicating that higher levels of conscientiousness were associated with better switching performance. This result fits the finding from Fleming et al. (2016), showing that conscientiousness was positively associated with mental set shifting, but not response inhibition or WM. This result suggests that the ability to flexible switch between tasks is especially associated with conscientiousness, whereas maintaining task goals and overcoming interference are not.

The result that conscientiousness influenced performance on the intelligence test as well as the flexibility task could indicate that higher levels of conscientiousness positively influenced performance on these tasks, possibly because more attentional control was allocated to these test situations. It is also possible that higher levels of conscientiousness affected the development of intelligence and cognitive flexibility or vice versa, but this has also to be clarified by longitudinal research.

Regarding the relations between EF and intelligence, we found significant associations between WM and intelligence in Experiment 1 as well as Experiment 2. This result is in line with previous research demonstrating relations between updating or WM and intelligence in children (Brydges et al., 2012) and adults (Benedek et al., 2014; Friedman et al., 2006).

Although the present results deliver an important insight into the relations between personality, EF, and intelligence, our study has some limitations that have to be considered: When interpreting associations of personality with EF or intelligence, poor reliabilities of the personality questionnaires have to be taken into account. In Experiment 1, we used a German short version of the HiPIC-30. Bleidorn and Ostendorf (2009) investigated psychometric properties of the German HiPIC in a sample of 223 students. The expected five-factor structure was replicated and reliability coefficients in the self-report version ranged from 0.82 (conscientiousness) to 0.88 (benevolence). However, children in their study were older (11–15 years) than the children in our study (9–10 years). Laidra et al. (2007) applied the Estonian Big Five Questionnaire for Children (EBFQ-C) via self-report in a sample of 7–11 years old students. In grades 2 and 3, they found comparable reliability coefficients ranging from 0.47 (extraversion) to 0.72 (conscientiousness). In Experiment 2, the reliabilities of the BFI-S scales were comparable to those reported in the longitudinal German Socio-Economic Panel Study (SOEP) with 1029 participants (Weinhardt & Schupp, 2014).

Moreover, the combination of hierarchical levels of personality factors, EF, and intelligence might have influenced the strength of their relations. Kretzschmar et al. (2018) showed that according to the Brunswik symmetry principle, the highest correlation between two constructs can be expected if constructs are investigated at a similar level. In their study, the correlations between personality factors and intelligence were substantially different depending on the combination of hierarchical levels. Especially Openness is a heterogeneous construct and the correlations with intelligence on the facet level differed significantly from no effect to a large effect. In our study, we applied two short personality inventories and thus, it was not possible to analyze relations on the facet level.

A further limitation is that WM, inhibition and cognitive flexibility were assessed by only one task. Especially in the light of the task-impurity problem (Denckla, 1994) it would be helpful to apply different tasks for each EF domain. Tasks measuring EF often require more than one EF. Furthermore, in EF tasks are always other, non-executive cognitive abilities such as processing speed, verbal ability or visuo-spatial ability involved (van der Sluis et al., 2007). Therefore, the relation between the performance on EF tasks and personality might be based on the executive or the non-executive demands of a task. In contrast to EF, intelligence was modeled as a latent variable in the SEM in Experiment 1 and Experiment 2. This discrepancy might have influenced the magnitude of the relations of personality with EF and intelligence (Marsh et al., 2009; Rhemtulla et al., 2019).

Moreover, we applied an intelligence test and EF tasks with time limits which could restrict the generalizability of our results. Eysenck (1959) showed that time limits moderated the association between extraversion and intelligence performance and there is evidence that pressure causes decrements in performance on cognitive and motor tasks (e.g., Beilock & Carr, 2005; Beilock et al., 2004; Masters, 1992). A prominent explanation for these decrements is the distraction hypothesis which proposes that pressure-filled situations distract attention away from the task, leading to poorer performance (Beilock & Carr, 2005; Beilock et al., 2004; Byrne et al., 2015). Byrne et al. (2015) investigated the moderating effects of personality factors on decision-making ability and performance under social and combined social and time pressure. They found that neuroticism and agreeableness negatively predicted performance under social pressure and combined social and time pressure. Moreover, time limits might have increased performance anxiety, especially in children. Although tasks were child-friendly, we cannot rule out the possibility that the general test situation or the time limit influenced the relations of neuroticism with intelligence in Experiment 1. Moutafi et al. (2006a, 2006b) showed that neuroticism was significantly correlated with intelligence for a group of high-anxiety adults but not for a low-anxiety group indicating that the relationship between neuroticism and intelligence might be mediated by test anxiety.

In sum, our findings deliver an important insight into the relations between personality, EF, and intelligence in children and young adults. Whereas neuroticism could constrain intelligence performance and inhibition abilities in children, conscientiousness could facilitate inhibition abilities in children as well as intelligence performance and cognitive flexibility in young adults.

## Data Availability

The data that support the findings of this study are available from the corresponding author, Verena E. Johann, upon reasonable request.

## Appendix A. Task and questionnaire specifications in Experiment 1

The tasks in Experiment 1 were adaptive (from level 1 to 7) and after a certain number of correct answers (see below) the difficulty level increased. Participants received informative feedback by means of a progress bar at the top of the screen. The progress bar turned green if participants pressed the correct key in time. After an incorrect or too slow reaction the progress bar turned red and after a certain number of incorrect answers (see below) the difficulty level was decreased.

### WM: complex span task (cf. Karbach et al., 2015)

The complex span task comprised 18 trials and 3 practice trials and started with a set size of 2 dinosaurs and 2 processing tasks. Trials consisted of two parts: At the encoding stage, a sequence of dinosaurs was presented without presentation time limit and participants were instructed to memorize them. After pressing the space bar, the background processing decision task appeared and participants had to decide if a star or a moon was presented. There was no presentation time limit for the processing task. After pressing the space bar again, the next dinosaur was presented. At the recall stage, all dinosaurs appeared and children were instructed to reproduce the sequence seen at the encoding stage in the correct order by subsequently clicking on the appropriate pictures. If the performance on both the processing and the recall task was correct on 5 successive trials, the number of to-be-remembered dinosaurs was increased by one in the next trial with a maximum of eight dinosaurs on level 7. If the performance on the processing or the recall task was incorrect two times in a row the number of dinosaurs was decreased by one.

### Inhibition: Stroop-like task (cf. Borella et al., 2010)

The Stroop-like task consisted of 130 trials and 17 practice trials. Congruent (50%) and incongruent trials were presented in random order. After ten correct trials, difficulty was increased by reducing the stimulus presentation time and the response time window. Difficulty was decreased after three incorrect or too slow reactions by increasing presentation time and response window. Stimuli were presented for 600 ms (level 1) to 500 ms (level 7) with a response window from 2250 ms (level 1) to 750 ms (level 7) depending on the current level and a response–stimulus interval (RSI) of 1000 ms.

### Flexibility: task switching (cf. Karbach & Kray, 2009)

The task comprised 7 mixed-task blocks (119 trials) as well as 34 practice trials. Visual stimuli consisted of 14 images of vegetables and 14 images of fruit. Each image was available in two sizes (small and big). Stimuli were presented for 1500 ms (level 1) to 800 ms (level 7) with a response window from 3400 (level 1) to 800 ms (level 7) depending on the current level and a RSI of 1000 ms. After ten correct trials, the difficulty was increased and decreased after three erroneous or too slow answers.

### Personality: HiPIC-30 (Bleidorn & Ostendorf, 2009; Vollrath et al., 2016)

We used a German short version of the Hierarchical Personality Inventory for children. Because there is no evaluated German short version, we selected those items from the German long version with 144 items (Bleidorn & Ostendorf, 2009) which are included in the English short version (HiPIC-30; Vollrath et al., 2016). The questionnaire comprised six items referring to neuroticism (e.g., “I am quick to worry about things”), six items referring to imagination (e.g., “I have a broad range of interests”), six items referring to conscientiousness (e.g.,“I finish tasks to the very end”), six items referring to benevolence (e.g.,“I am easily incensed by things”) and six items referring to extraversion (e.g., “I talk to people easily”).

## Appendix B. Task and questionnaire specifications in Experiment 2

### WM: counting span task (cf. Kane et al., 2004)

In the counting span task, participants recalled digits against a background counting task. Each stimulus consisted of three to nine dark blue circles, and one, three, five, seven, or nine dark blue squares, and one to five light green circles on a gray background. Participants were to count the number of dark blue circles in each display and repeat the total number. There was no time limit but participants were instructed to count continuously. The experimenter then pressed a key and another display or the recall cue appeared. At the recall cue, participants had to recall the respective numbers of dark blue circles in the correct order. Set sizes ranged from two to five items, with a total of eight sets. The task started with three practice items.

### Inhibition: Flanker task (cf. Eriksen & Eriksen, 1974)

Participants performed 20 practice trials followed by five experimental blocks (200 trials). The presentation order of the stimuli was randomized within blocks. Stimuli were presented for 200 ms without a time limit but participants were instructed to respond as quickly and accurate as possible.

### Flexibility: Task switching (cf. Karbach & Kray, 2009)

The task comprised 6 mixed-task blocks (102 trials) as well as 34 practice trials. Visual stimuli consisted of 18 images of vegetables and 18 images of fruit. Each image was available in two sizes (small and big). Stimuli were presented for 5000 ms with a response window of 5000 ms.

### Personality: BFI-S (Gerlitz & Schupp, 2005)

The questionnaire comprised three items referring to neuroticism (e.g., “I am quick to worry about things”), three items referring to openness (e.g., “I have a vivid imagination”), three items referring to conscientiousness (e.g., “I work thoroughly”), three items referring to agreeableness (e.g., “I treat people with care and respect”) and three items referring to extraversion (e.g., “I am communicative”).

## Code availability

The software application that supports the findings of this study is available from the corresponding author, Verena E. Johann, upon reasonable request.
